# The psychological future of unemployed individuals and workers: invariance measurement model and mean differences

**DOI:** 10.1007/s12144-023-04565-6

**Published:** 2023-03-29

**Authors:** Vinicius Coscioni, Catarina Luzia de Carvalho, Maria do Céu Taveira, Ana Daniela Silva

**Affiliations:** School of Psychology, University of Minho, Braga, Portugal

**Keywords:** Psychological future, Life project, Future time orientation, Unemployed individuals, Factor analysis, Invariance measurement

## Abstract

This study compared the psychological future of unemployed individuals and workers during the Covid-19 pandemic. It used the data from two previous data collections, one with unemployed individuals and another with workers. Participants from the two datasets were paired considering the same gender and similar age and education degree. The analyzed sample consisted of 352 participants, of which 176 were unemployed individuals and 176 were workers. The psychological future was measured by the Future Time Orientation Scale and the Life Project Scale. Both scales fit the sample of unemployed individuals and were invariant at the metric level across occupation status. Partial scalar model met good fit after freeing the intercepts of one item in each scale. In contrast to the hypothesis, compared to workers, unemployed individuals did not have lower rates in the assessed features of their psychological future. Conversely, for some variables, the rates were even higher among unemployed individuals. Unexpected results and limitations are discussed.

**Supplementary Information:**

The online version contains supplementary material available at 10.1007/s12144-023-04565-6.

## Introduction

The economic crisis caused by the Covid-19 pandemic has impacted people worldwide (Santilli et al., 2021). The economic recession has affected many sectors of society, with a large escalation in unemployment rates (Instituto de Emprego e Formação Profissional [IEFP], 2021). Unemployment is associated with economic, social, and psychological vulnerability (Drosos et al., 2021), impacting how individuals deal with their future (Bonanomi & Rosina, 2022; Taveira et al., 2023). Moreover, future-oriented people tend to perceive themselves as more employable (Chen et al., 2022) and have less risk of being not in employment, education, or training (Cheng & Nguyen, 2022). Thus, optimizing the psychological future of unemployed individuals may help improve their quality of life and enhance their chances in job search. Previous research has contrasted the psychological future of unemployed individuals, workers, and students, with the unemployed group being less future-oriented (Guthrie et al., 2009; Parola & Donsì, 2019). Nevertheless, comparisons of the psychological future of people with different working status during the Covid-19 pandemic have not still yet been made. This would help elucidate the impact of such a period of crisis on how unemployed individuals deal with their future. This study compares the psychological future of unemployed individuals and workers during the Covid-19 pandemic.

### Psychological future

How people perceive their future has an impact on how they behave in the present. One of the main exponents of this idea was Lewin (1951), who stated the psychological future (i.e., one’s perceptions regarding the future) is a key component of human motivation. After Lewin, countless theories have described different aspects of people’s psychological future (Seginer, 2009). According to Coscioni et al. (2020), those theories refer to distinct psychological constructs that focus on different facets of people’s psychological future. Even though those constructs tend to be correlated one to the other (Jia et al., 2022; Kačmár & Beere, 2021; van Hooft, 2018), they focus on different facets of one’s perceptions regarding the future. In this article, two constructs of the psychological future are examined, i.e., future time orientation (FTO) and life project (LP).

According to Gjesme (1983), FTO is “a general capacity to anticipate, shed light on and structure the future, including a cognitive elaboration of plans and projects and reflecting the degree of concern, involvement and engagement in the future” (p. 452). Gjesme distinguished FTO and future orientation; the former being a personality trait, whereas the latter entails the manifestations of FTO in specific tasks. After Gjesme (1983), other theories described FTO as a disposition rather than a trait (Husman & Shell, 2008; Zimbardo & Boyd, 1999). Those theories pictured FTO as a relatively enduring personal feature, yet one more influenced by the current situation than a trait. A personality trait is supposedly less impacted by the circumstances of adult life. Based on this perspective, Coscioni et al. (2021b) defined FTO as the disposition to have current psychological functioning impacted by the psychological future.

Different measures have been created to assess dispositions of the psychological future. The Future Time Perspective Inventory (Heimberg, 1963) is a unidimensional measure. In turn, the Zimbardo Time Perspective Inventory (Zimbardo & Boyd, 1999), the Consideration of Future Consequences Scale (Strathman et al., 1994), the Time Orientation Scale (Holman & Silver, 1998) and the *Inventário de Perspectiva Temporal* (Janeiro, 2012) are examples of multidimensional measures comparing the past, present, and future orientations. To our knowledge, only three measures assess dispositions of the psychological future multidimensionally, the Future Time Orientation Measure (Gjesme, 1979), the Future Time Perspective Scale (Husman & Shell, 2008), and the Future Time Orientation Scale (FTOS; Coscioni et al., 2021b), with the latter being an attempt to solve limitations of the two first ones. The FTOS has two dimensions: (a) distance, i.e., perception of distance into the future; and (b) impact, i.e., the influences of the psychological future in current decisions and behavior.

In turn, LP is defined by Coscioni (2021) as “an ongoing evolving process to form, enact, and maintain intentional structures and actions that, altogether, encompass a long-term, meaningful, and prospective narrative capable of guiding decisions and behavior in daily life” (Coscioni, 2021, p. 144). A LP entails a personal construction of one’s intended future, a set of intentional structures and actions toward the future. Therefore, a LP is a state, a specific manifestation of FTO in a specific and important task of daily life, i.e., life planning.

LPs have been mostly assessed via qualitative methods (Coscioni, 2021). Yet, examples of LP measures are the Life Project Reflexivity Scale (Di Fabio et al., 2018) and the Life Project Scale (LPS; Coscioni et al., 2021b). The former assesses LP reflexivity through three dimensions: (a) authenticity, i.e., congruence between LP and personal values; (b) acquiescence, i.e., the passive integration of societal values into one’s LP; and (c) clarity, i.e., awareness of one’s LP. The LPS assesses LP coherence through two dimensions: (a) identification, i.e., clearness regarding one’s intended future; and (b) involvement, i.e., the enactment of plans and efforts toward one’s intended future.

### The psychological future of unemployed people

Most adults are concerned about their future careers and ascribe an important role to their work identities (Park et al., 2018). Thus, being employed is associated with important psychological resources, whereas job loss may lead to a detriment of quality of life (Taveira et al., 2017). For unemployment is an undesirable and uncontrolled event (Panari & Tonelli, 2022), it can change one’s perceptions of the future (Fidelis & Mendonça, 2021). Indeed, long-term unemployment produces a corrosive, pervasive, and persistent effect on individuals, increasing the difficulty in planning for the future and exploring new possible future work (Mühlhaus et al., 2021; Zacher, 2013). Moreover, past unemployment experiences are predictors of subjective likelihood of unemployment risk in the future, driving insecurity and unhappiness (Knabe & Rätzel, 2011).

Previous research has compared the psychological future of unemployed individuals, students, and workers, with unemployed individuals being less future-oriented (Guthrie et al., 2009; Parola & Donsì, 2019). Moreover, future orientation has been pointed out as a moderator of the relationships between working status and mental health indicators (Parola & Marcionetti, 2022), and a mediator of the relationship between working status and well-being (Felaco & Parola, 2022). Despite the evidence of reduced future orientation, qualitative studies have described a few positive envisions of the future among employed individuals, especially youths who reported shorter periods of unemployment (Mühlhaus et al., 2021).

The psychological future of unemployed individuals in periods of economic crisis have been the subject of at least two studies, both in Greece during the World economic crisis initiated in 2008. One study was based on 39 interviews and revealed most participants were pessimistic about their future. However, their prospective life narratives frequently referred to alternative goals and future actions to achieve a work position and better living conditions (Daskalaki & Simosi, 2018). The other study was based on a focus group with seven individuals. The study mostly emphasized the constraints of the economic crisis in the construction of participants’ narratives toward the future (Sools et al., 2017).

Optimizing the psychological future of unemployed individuals must be a priority in employment programs. According to Patel et al. (2020), the assessment of employability programs should include non-economic indicators, such as self-esteem, self-efficacy, and future orientation. Many studies investigating the effects of employability programs and career counseling have encompassed future-related variables in their tests (Ginevra et al., 2017) including studies in the context of the Covid-19 pandemic (Santilli et al., 2021). Nevertheless, to our knowledge, no previous research has examined the psychometric properties of measures of the psychological future among unemployed individuals.

### The current study

This study compares the psychological future of unemployed individuals and workers during the Covid-19 pandemic. The two first specific goals are: (1) to assess the internal structure and reliability of the FTOS and the LPS among unemployed individuals; and (2) to test the invariance of the two measures across unemployed individuals and workers. Both measures have already been tested in invariance models that identified they are equivalent, at the scalar level, across students and workers (Coscioni et al., 2021b). Even though the results endorse the scales are invariant across occupational statuses, the equivalence across people with and without an occupation is still not tested. Therefore, testing the internal structure and reliability of the two measures would provide evidence on the use of such measures with unemployed individuals. In addition, invariance models across workers and unemployed individuals would allow for safer comparisons across those groups. This is particularly relevant considering that unemployment may impact how one deals with the future (Bonanomi & Rosina, 2022). Being the two measures invariant across students and workers as well as across gender, culture, education, and age (Coscioni et al., 2021b), it seems reasonable to assume they would also fit samples of unemployed individuals. Therefore, our hypotheses are: (1) the FTOS and the LPS will fit the sample of unemployed individuals; and (2) the FTOS and the LPS will be invariant across unemployed individuals and workers.

The third specific goal is (3) to compare the FTO and LP of unemployed individuals and workers during the Covid-19 pandemic, considering the main effects and interaction terms of genders and education. The psychological future of unemployed individuals and workers have already been compared in two previous studies, both before the Covid-19 pandemic (Guthrie et al., 2009; Parola & Donsì, 2019). Considering that the pandemic incited an economic crisis worldwide (Santilli et al., 2021) and affected people’s orientation toward the future (Ceccon & Moscardino, 2022; Lenzo et al., 2022), it is relevant to examine differences across workers and unemployed individuals in this particular time. Moreover, the two previous studies did not control for the effect of gender and education, variables that may impact people’s psychological future (Rudolph et al., 2018; Salgado & Berntsen, 2018). Considering the previous results of those studies, the third hypothesis is: (3) compared to workers, unemployed individuals will have lower rates of FTO and LP.

This study is not a replication of the works of Guthrie et al. (2009) and Parola and Donsì (2019). Rather, it includes two measures of the psychological future not previously compared across unemployed individuals and workers. The use of different measures is not an attempt to improve the study design but an effort to assess other facets of the psychological future still not compared across unemployed individuals and workers. Guthrie et al. (2009) used the theoretical approach of Zimbardo and Boyd (1999), which contains a general futural factor related to presence of goals, future planning, and punctuality. In turn, Parola and Donsì (2019) used a single item measuring future planning. In this study, a disposition to be impacted by the psychological future (i.e., FTO) and the coherence of the intended future (i.e., LP) were assessed. Therefore, the three studies are not measuring the same phenomenon yet phenomena of the same class.

## Method

### Participants and procedures

This study compares two datasets: one from the Careers Project (ALG-06-4234-FSE-000047; Silva & Taveira, 2021) and another from the Future Time Orientation and Life Project: A Theoretical and Transcultural Approach from a Psychosocial Perspective (FTOLP; Coscioni et al., 2021a). The Career Project is a partnership for social impact to support employability in Algarve, a tourism-dependent region located in the southside of Portugal. Algarve is the region in Portugal that has witnessed the largest increase in unemployment rates during the pandemic (PORDATA, 2021). Over 33 thousand residents in Algarve have become unemployed during the pandemic, representing an increase of 150% (IEFP, 2021). Participants from the Careers Project were unemployed individuals invited by a governmental institution to participate in a career intervention. Those interested in the intervention filled in an online survey, from April to May 2022. In turn, the Project FTOLP consisted in an online data collection from April to December 2020, which included participants with different working status. For this study, we considered only those participants who were working. Participants from both studies provided their consent form before answering the survey and the research procedures were approved by ethical commissions from Portugal.

We randomly formed pairs of participants from the two studies considering the same gender, education (with or without a college degree), nationality (Portuguese citizens or foreigners), and age (not older than five years). In total, 176 pairs could be formed. Therefore, the final sample consisted of 354 participants aged from 19 to 67 years old (*M* = 40.9, *SD* = 9.92), predominantly female (*n* = 264, 75.0%), from Portugal (*n* = 286, 81.3%), and without a college degree (*n* = 180, 51.1%). Among unemployed individuals, the mean time without working was 1.5 years (*SD* = 1.72) and nearly four fifths (*n* = 140, 79.5%) became unemployed during the pandemic.

### Measures

#### Future time orientation scale (FTOS; appendix 1)

The FTOS was originally created in Portuguese (both European and Brazilian) and English (Coscioni et al., 2021b). It contains two subscales that measure impact (five items, e.g., “I value activities that may benefit me in the long run”) and distance (three items, e.g., “Two years in the future seems to me like a short period of time”). Each statement is responded to in a 7-point scale ranging from ‘strongly disagree’ to ‘strongly agree.’

#### Life Project Scale (LPS; appendix 2)

The LPS was originally created in Portuguese (both European and Brazilian) and English (Coscioni et al., 2021b). The scale contains two subscales that measure identification (four items, e.g., “I am aware of what I want for my future life”) and involvement (four items, e.g., “I’m making efforts to achieve what I want for the future”). Items are responded to in a 7-point scale ranging from ‘totally disagree’ to ‘totally agree.’

### Data analysis

Confirmatory factor analyses (CFA) were implemented to assess the internal structure of the FTOS and the LPS across the two groups and in the entire sample. Maximum likelihood robust (MLR; Satorra & Bentler, 2001) estimator was used because the response patterns of both scales violated multivariate normality in all conditions (Appendix 3). In addition, MLR outperforms ordinal estimators when testing scales with six to seven response categories (Rhemtulla et al., 2012). The following fit indices and cutoffs assessed the models’ goodness of fit: comparative fit index (CFI) and Tucker-Lewis index (TLI) above 0.95, and root mean square error approximation (RMSEA) and standardized root mean residual (SRMR) equal or below 0.08 (Schreiber et al., 2006). Alternatively, the following cutoffs were considered acceptable: 0.90 ≤ *CFI* < 0.95, 0.90 ≤ *TFI* < 0.95, 0.080 ≥ *RMSEA* > 0.100, and 0.080 > *SRMR* ≥ 0.100 (Brown, 2006). Mahalanobis distance was computed and suggested 5 to 14 outliers across subsets and scales. CFA were implemented with and without outliers. No big differences were observed after the removal of outliers and, thus, they were retained.

Multigroup CFA were performed to assess the invariance of the factor structure (configural model), factor loadings (metric model), and intercepts (scalar model) across unemployed persons and workers. Invariance was tested by scaled chi-squared difference tests (Satorra & Bentler, 2001) comparing configural and metric models, and metric and scalar models. We expected differences in RMSEA and CFI across models below or equal to 0.005 and above or equal to − 0.010, respectively (Cheung & Rensvold, 2002).

Reliability was assessed by Cronbach’s alpha (*α*), McDonals’ omega (*Ω*), and average variance extracted (AVE). The following *α* and *Ω* cutoffs were used: values below 0.50 are inacceptable; between 0.51 and 0.60 are poor; between 0.61 and 0.70 are questionable; between 0.71 and 0.80 are moderate; between 0.81 and 0.90 are good; above 0.91, excellent (Gliem & Gliem, 2003). AVE values above 0.50 were expected (Fornell & Larcker, 1981).

Mean differences across groups were assessed via factorial multivariate analysis of variance (MANOVA) considering four dependent variables, i.e., the FTOS and LPS’s subscales, and three independent variables, i.e., working status, gender, and education. Bootstrap (500 resampling; 95% CI BCa) was implemented to correct violations of univariate and multivariate normality and homogeneity of covariances (Appendix 4). Factorial univariate analysis of variance (ANOVA) and post-hoc t tests with Hochberg correction were carried out to assess significant results. The magnitude of differences was measured by *d* of Cohen with bootstrap correction (500 resampling; 95% CI BCa). The following cutoffs were used for interpretation: |*d*| < 0.2, negligible; |*d*| < 0.4, small; |*d*| < 0.8, medium; otherwise, large (Cohen, 1988).

The sample size was appropriate for all analyses. For the CFA, a sample size calculator (Soper, 2022) suggested the minimum of 100 participants for model structure. In addition, considering *α* = 0.05, *β* = 0.80, and a sample size of and 176 participants, CFA can detect significant parameters with an effect of 0.22. As for the MANOVA, two cells had 17 participants (i.e., male unemployed persons with a college degree and male workers with a college degree), less than the minimum of 20 suggested by Hair et al. (2006). However, all cells had more participants than the number of dependent variables (*n* > 4). In addition, considering *α* = 0.05, *β* = 0.80, and a sample size of 352, a factorial MANOVA 2 × 2 × 2 can detect significant effects at part. η^2^ = 0.017 (Erdfelder et al., 1996).

All analyses were carried out in the software R 4.1.1 (R Core Team, 2021). CFA were tested in lavaan 0.6-9 (Rosseel, 2012). Reliability was assessed in semTools 0.5-5 (Jorgensen et al., 2021). Correlations were computed with psych 2.3.3 (Revelle & Revelle, 2015). MANOVA with bootstrap correction was implemented in MANOVA.RM (Friedrich et al., 2018).

## Results

**Fig. 1:**
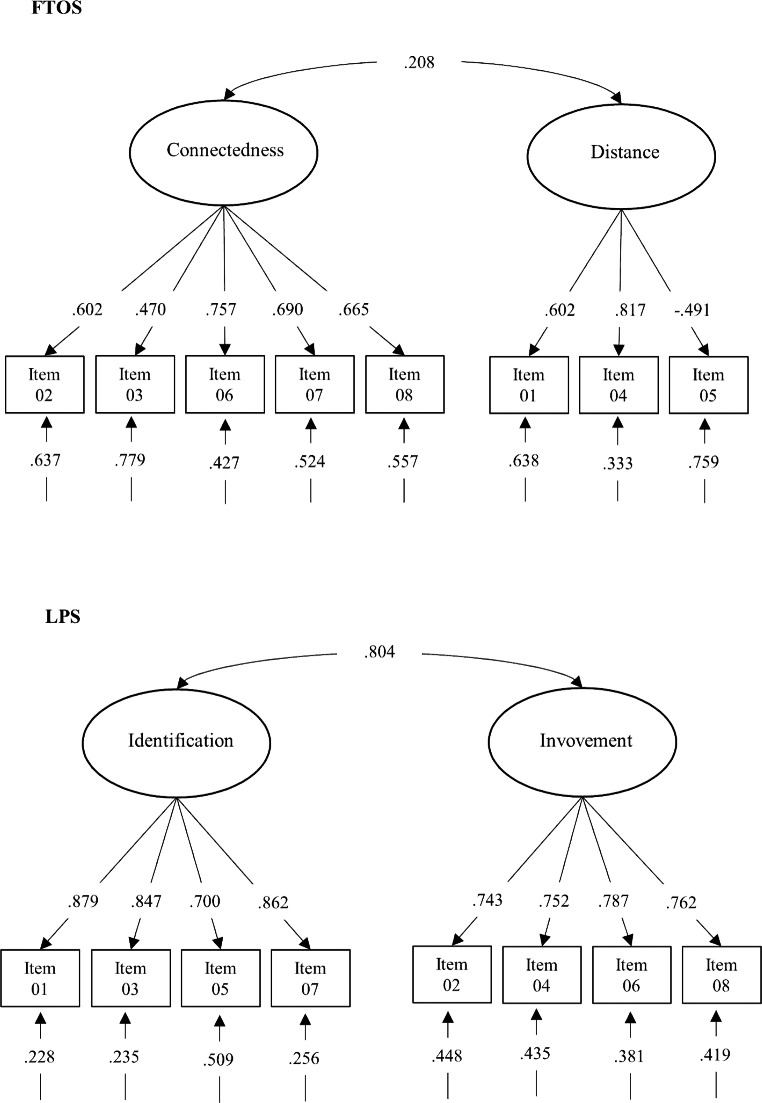
CFA with unemployed individuals

Table 1 exhibits the fit indices of CFA and invariance models. The FTOS met excellent fit indices in all subsets, except for TLI, which reached acceptable results among unemployed individuals and in the general sample. The LPS met excellent fit indices among unemployed individuals and in the general sample. However, among workers, RMSEA, CFI, and TLI resulted in acceptable values. As for the invariance measurement models, both scales met partial scalar invariance with the intercepts of item 2 (in both scales) being higher among workers. Figure 1 exhibits the factors loadings, correlations between factors, and residual variances of the retained models among unemployed individuals. All factor loadings are higher than 0.50, except for items 3 and 4 of the FTOS. Correlations between the FTO dimensions are non-significant. In turn, correlations between the LP dimensions are strong.

**Table 1:** CFA

	χ^2^(df)	RMSEA[90% CI]	CFI	TLI	SRMR	Sc. χ^2^(df)	ΔRMSEA	ΔCFI
Internal structure: FTOS								
unemployed	28.4(19)	0.053 [0.000; 0.089]	0.957	0.937	0.054			
workers	24.4(19)	0.040 [0.000; 0.080]	0.974	0.961	0.055			
all	34.1(19)*	0.047 [0.022; 0.070]	0.962	0.944	0.041			
Internal structure: LPS								
unemployed	33.9(19)*	0.067 [0.036; 0.095]	0.971	0.958	0.039			
workers	49.8(19)**	0.096 [0.069; 0.124]	0.934	0.902	0.069			
all	39.7(19)*	0.056 [0.036; 0.075]	0.977	0.966	0.043			
Invariance: FTOS								
configural	82.9(38)**	0.082 [0.062; 0.102]	0.955	0.933	0.049			
metric	87.8(44)**	0.075 [0.056; 0.094]	0.955	0.943	0.060	5.0(6)	− 0.007	0.000
scalar	107.4(50)**	0.081 [0.063; 0.098]	0.942	0.935	0.065	22.7(6)**	0.006	− 0.013
partial^1^	100.1(49)**	0.077 [0.059; 0.095]	0.948	0.941	0.062	13.1(5)*	0.002	− 0.007
Invariance: LPS								
configural	52.9(38)	0.047[0.007; 0.074]	0.965	0.948	0.049			
metric	62.4(44)*	0.049[0.016; 0.073]	0.957	0.945	0.058	9.4(6)	0.002	− 0.008
scalar	84.2(50)*	0.062[0.040; 0.084]	0.920	0.910	0.065	23.7(6)**	0.013	− 0.037
partial^1^	70.0(49)*	0.049[0.021; 0.072]	0.951	0.944	0.060	7.6(2)	0.000	− 0.006

Notes. n = 176, for unemployed persons; and n = 176, for workers, *significant at α = 0.05, **Significant at α = 0.001, FTOS = Future Time Orientation Scale, LPS = Life Project Scale, 1Intercepts of item 2 not fixed; partial model compared to metric model

Table 2 displays the factors’ descriptive statistics and reliability. The LPS met good reliability in all conditions, whereas the FTOS met acceptable to moderate reliability. Both FTO dimensions reached low AVE in all conditions.

**Table 2:** Descriptive statistics, reliability, and Spearman correlations

	unemployed				workers				all			
	M(SD)	α	Ω	AVE	M(SD)	α	Ω	AVE	M(SD)	α	Ω	AVE
FTOS												
Dis	4.4(1.13)	0.66	0.67	0.41	4.5(1.20)	0.68	0.73	0.50	4.4(1.17)	0.67	0.70	0.45
Imp	5.9(0.70)	0.77	0.77	0.41	5.6(0.73)	0.74	0.75	0.38	5.7(0.72)	0.76	0.76	0.39
LPS												
Idn	5.4(1.21)	0.90	0.90	0.70	5.4(0.98)	0.83	0.85	0.59	5.4(1.10)	0.87	0.88	0.65
Inv	5.4(1.06)	0.84	0.84	0.58	5.2(1.10)	0.85	0.85	0.60	5.3(1.08)	0.84	0.85	0.58

Notes. n = 216, *p > .050, ***p > .001, FTOS = Future Time Orientation Scale, Dis = distance, Imp = impact, LPS = Life Project Scale, Idn = identification, Inv = involvement

Table 3 shows the results of MANOVA and univariate ANOVA. Pillai’s V was significant for working status and the interaction term of working status and education. After bootstrap correction, only the interaction term remained significant. Univariate ANOVA showed significant differences in impact across working status and the interaction term of working status and gender. However, only the interaction term remained significant after bootstrapping. The interaction term of working status and education was significant for identification and involvement, even though involvement did not remain significant after bootstrap correction. Gender was significant for involvement but only after bootstrapping.

**Table 3:** MANOVA

						Bootstrap correction	
Multivariate test	Pillai V	df1	df2	p	part. η2	MATS	p
intercept	0.000	4	341	1.00			
working status	0.033	4	341	0.023*	0.033	2.546	0.592
gender	0.014	4	341	0.302	0.014	9.866	0.078
education	0.005	4	341	0.770	0.005	0.742	0.908
working status*gender	0.017	4	341	0.209	0.021	8.933	0.088
working status*education	0.040	4	341	008*	0.040	11.349	0.024*
gender*education	0.008	4	341	0.631	0.008	3.474	0.434
working status*gender*education	0.005	4	341	0.803	0.005	1.507	0.782
						Bootstrap correction	
Univariate tests	F	df1	df2	p	part. η2	MATS	p
Distance							
Intercept	0.000	1	344	1.000			
working status	0.094	1	344	0.759	0.000	0.279	0.570
gender	1.102	1	344	0.295	0.003	1.426	0.274
education	0.002	1	344	0.967	0.000	0.177	0.676
working status*gender	0.691	1	344	0.406	0.002	0.533	0.458
working status*education	0.418	1	344	0.519	0.001	0.001	0.984
gender*education	0.735	1	344	0.392	0.002	0.775	0.406
working status*gender*education	1.228	1	344	0.269	0.004	1.295	0.262
Impact							
intercept	0.000	1	344	1.000			
working status	7.640	1	344	0.006*	0.022	1.370	0.248
gender	0.125	1	344	0.724	0.000	0.298	0.564
education	0.473	1	344	0.492	0.001	0.017	0.896
working status*gender	5.765	1	344	0.017*	0.016	8.196	0.002*
working status*education	2.742	1	344	0.099	0.008	2.534	0.896
gender*education	1.837	1	344	0.176	0.005	2.323	0.140
working status*gender*education	0.000	1	344	1.000	0.000	0.000	1.000
Identification							
intercept	0.000	1	344	1.000			
working status	0.013	1	344	0.909	0.000	0.010	0.912
gender	3.632	1	344	0.058	0.010	3.691	0.062
education	0.482	1	344	0.488	0.001	0.419	0.526
working status*gender	0.080	1	344	0.777	0.001	0.000	0.972
working status*education	7.844	1	344	0.005*	0.022	5.506	0.008*
gender*education	0.000	1	344	0.989	0.000	0.000	0.986
working status*gender*education	0.172	1	344	0.679	0.000	0.199	0.638
Involvement							
intercept	0.000	1	344	1.000			
working status	1.498	1	344	0.222	0.004	0.887	0.368
gender	3.499	1	344	0.062	0.010	4.451	0.030*
education	0.002	1	344	0.965	0.000	0.129	0.748
working status*gender	0.413	1	344	0.521	0.001	0.203	0.678
working status*education	4.034	1	344	0.045*	0.012	3.309	0.072
gender*education	0.314	1	344	0.576	0.001	0.376	0.554
working status*gender*education	0.011	1	344	0.917	0.000	0.013	0.922

Notes. n = 352, *significant at α = 0.05, **Significant at α = 0.001, MATS = modified ANOVA-type statistic

Table 4 presents the results of post-hoc t tests. A small difference across working status was detected for impact, with unemployed participants being more future-oriented. Considering the interaction term with gender, the difference increased among females, with a medium magnitude being detected. As for the interaction term of working status and education, a medium difference in identification across educational degrees was detected among workers, with participants without a college degree having clearer LPs. In addition, a small difference in involvement across working status was detected among participants with a college degree, with unemployed participants being more involved with their LPs. Differences across gender in involvement were not detected in the t test.

**Table 4:** Post-hoc tests

	Group 1^1^			Group 2^1^			t test					Bootstrap	
	n	M	SD	n	M	SD	t	df	p	adj. p^1^	d	lower	upper
working status → impact													
unemployed X worker	176	0.08	0.54	176	-0.08	0.56	2.74	350	0.006*	---	0.29	0.11	0.52
working status*gender → impact													
female unemployed X female worker	132	0.12	0.54	132	-0.13	0.60	3.44	262	0.001**	0.002**	0.42	0.19	0.64
male unemployed X male worker	44	-0.02	0.53	44	0.06	0.40	-0.82	86	0.415	0.487	− 0.17	− 0.57	0.28
female unemployed X male unemployed	132	0.12	0.54	44	-0.02	0.53	1.49	174	0.143	0.298	0.26	− 0.06	0.60
female worker X male worker	132	-0.13	0.60	44	0.06	0.40	-2.33	174	0.057	0.158	− 0.36	− 0.65	− 0.07
working status*education → identification													
unemployed no degree X worker no degree	90	-0.11	1.24	90	0.20	0.77	-2.02	178	0.045*	0.108	− 0.30	− 0.59	− 0.01
unemployed degree X worker degree	86	0.10	0.99	86	-0.20	1.00	1.97	170	0.051	0.0.54	0.30	0.00	0.61
unemployed no degree X unemployed degree	90	-0.11	1.24	86	0.10	0.99	-1.21	174	0.227	0.180	− 0.18	− 0.48	0.14
worker no degree X worker degree	90	0.20	0.77	86	-0.20	1.00	3.00	174	0.003**	0.009**	0.45	0.17	0.73
gender → involvement													
female X male	264	-0.06	1.09	88	0.18	0.94	-1.87	350	0.062	---	− 0.24	− 0.44	0.01
working status*education → involvement													
unemployed no degree X worker no degree	90	-0.04	1.25	90	0.05	0.93	-0.58	178	0.565	0.568	0.09	− 0.37	0.22
unemployed degree X worker degree	86	0.18	0.98	86	-0.20	1.14	2.32	170	0.022*	0.020*	0.35	0.07	0.64
unemployed no degree X unemployed degree	90	-0.04	1.25	86	0.18	0.98	-1.32	174	0.188	0.181	− 0.20	− 0.51	0.12
worker no degree X worker degree	90	0.05	0.93	86	-0.20	1.14	1.61	174	0.109	0.113	0.24	− 0.02	0.54

Notes. n = 352, *significant at α = 0.05, **Significant at α = 0.001, 1Group 1 is the group on the left, whereas Group 2 is the group on the right. For instance, in the comparison “unemployed X worker” (second line), Group 1 is composed of unemployed individuals and Group 2 is composed of workers.2 Hochberg post-test

## Discussion and conclusions

This study compared the psychological future of unemployed individuals and workers during the Covid-19 pandemic. It began by assessing the internal structure of the FTOS and the LPS among unemployed individuals. Both scales properly fit the data. However, the AVE of both FTO dimensions was below 0.50, which is in line with previous research (Coscioni et al., 2021b). This means the FTOS’s items explain more errors than the variances in the constructs being measured. Despite such limitations, the other coefficients suggest the FTOS is a reliable measure and thus may be used with unemployed individuals.

The two measures have their invariance tested across unemployed individuals and workers. Unexpectedly, multigroup CFA detected invariance only at the metric level. This means the two scales have the same factor structures and factor loadings across working status but differences at the item intercept level. Partial scalar models indicate higher intercepts among workers in the item 2 of both measures. Thus, compared to unemployed individuals, workers were biased to endorse higher response categories. The LPS’s item 2 refers to time effort, i.e., “I’m spending a great deal of time on actions related to my future goals.” The higher intercept among workers might be related to the fact that they are expectedly more engaged with their careers since work positions often take great part in the weekly schedule. Conversely, the intercept difference in item 2 of the FTOS (i.e., “When making decisions, I think carefully about how my choices may influence the future”) still needs further investigation.

The scores in the four subscales were compared across unemployed individuals and workers, considering the direct effect and interaction terms of gender and education. Multivariate differences were detected only for the interaction term of working status and education. However, univariate ANOVA and post-hoc tests suggested differences in identification across education but only among workers. The results contrast to the existing literature (Guthrie et al., 2009; Parola & Donsì, 2019) and refute the hypothesis according to which unemployed individuals were expected to have lower rates in FTO and LP. The unexpected results might be related to the fact that this study embraces facets of the psychological future not assessed in the studies of Guthrie et al. (2009) and Parola and Donsì (2019). Nevertheless, even though those variables are not the same, future-related variables tend to be related one to the other among unemployed individuals (van Hooft, 2018) and other types of samples (Jia et al., 2022; Kačmár & Beere, 2021). Thus, we have no reason to accept different conclusions based on this justification.

Three other reasons might better justify the unexpected results. First, the unemployed individuals from the Careers Project responded to a survey after accepting an invitation to take part in career interventions. Therefore, higher rates in the psychological future might be expected. Second, most participants became unemployed during the pandemic, with the majority being jobless for less than one year. The existing literature suggests a sharper effect of long-term employment (Mühlhaus et al., 2021) and, thus, different results could have been found with a sample of individuals unemployed for a longer period of time. Last but not least, the hypothesis of lower rates in future-related variables among unemployed individuals is grounded on the assumption that unemployment is caused and maintained due to personal features. However, as discussed by Daskalaki and Simosi (2018), this assumption is a neoliberal construction that dismisses the effects of precarious employment.

Other mean differences were initially significant yet lost significance after bootstrap correction. Thus, the results may be biased due to violations of statistical assumptions. Unemployed individuals were more future-oriented and the difference was larger among females. In addition, unemployed individuals with a college degree were more involved with their LPs compared to workers with a college degree. Hence, the results are in the opposite direction of the hypothesis. One may suspect unexpected differences might be due to intercept differences. However, intercepts were higher among workers, meaning unemployed individuals had higher scores even with a bias of endorsing lower response categories. The interaction term of gender and education still needs further investigation, be that in studies with big samples or using qualitative methods.

This study has an important limitation, namely, the compared datasets are from two studies with data collections in different stages of the Covid-19 pandemic. The Project FTOLP occurred from April to December 2020, whereas the Careers Project took place from April to May 2022. Therefore, differences might be the result of the isolation measures of which participants from the first project were being targeted (Ceccon & Moscardino, 2022; Lenzo et al., 2022). Nevertheless, in the Project FTOLP, both the FTOS and LPS were invariant across Covid-related concern and impact (Coscioni et al., 2021b).

Despite limitations, this study brought about important contributions to understanding the psychological future of unemployed individuals. First, the analysis of the internal structure and reliability of the FTOS and LPS among employed individuals endorse the potential use of such measures with this group of the population. Second, invariance measurement models indicate differences in the psychological future of unemployed individuals and workers may be biased due to intercept differences. However, as in both scales only one item intercept was invariant across groups, the bias does not seem to deeply impact comparisons. Lastly, mean comparisons across groups are not in line with the initial hypothesis that unemployed individuals would have lower rates in FTO and LP. These results evidence unemployment might not always impact how individuals deal with their future after job loss. Considering that most participants were freshly unemployed, future-oriented activities could have been initiated in order to optimize job search (like, for instance, attending a career intervention).

In practical terms, in career interventions, there might be cases in which unemployed clients’ rates in future-related variables are even higher compared to employed ones. Among those less future-oriented and with an unclear LP, the optimization of the psychological future still may enhance their self-confidence on future employment (Chen et al., 2022) and decrease the risk of not being in employment, education, or training (Cheng & Nguyen, 2022). Consequently, it would be important to pursue an initial assessment of clients’ psychological future. This might effectively allow career counselors to distinguish individuals who would actually benefit from interventions aimed at the consolidation of the psychological future. In this context, special attention should be given to both individual and contextual needs for returning to the labor market.

*Notes. n =* 176.

## Electronic supplementary material

Below is the link to the electronic supplementary material.

Supplementary Material 1

## Data Availability

This study refers to datasets from two previous data collections. The use of the datasets was authorized. The analyzed dataset may be accessed upon reasonable request.
