# Supplementary material for: The psychological future of unemployed individuals and workers: invariance measurement model and mean differences
Source: Curr Psychol. 2023 Mar 29:1–12. Online ahead of print. doi: 10.1007/s12144-023-04565-6 (PMC10052292; doi:10.1007/s12144-023-04565-6)
Supplement: Supplementary file 1 — Supplementary Material 1 [file 12144_2023_4565_MOESM1_ESM.docx]

**Appendix 1**

Future Time Orientation Scale – items translated from Portuguese to English

(The Portuguese form may be accessed via contact with the corresponding author)

1) Two years in the future seems to me like a short period of time

2) When making decisions, I think carefully about how my choices may influence the future

3) I value activities that may benefit me in the long run

4) For me, something that will happen two years from now seems relatively soon

5) When I think of something that will happen two years from now, I feel like there is a great deal of time ahead

6) When I want something, I think carefully about what I have to do to achieve it in the future

7) I make connections between the things I do now and what may happen with me in the future

8) I make sacrifices in the present if I think they may benefit me in the future

**Appendix 2**

Life Project Scale – items translated from Portuguese to English*

(The Portuguese form may be accessed via contact with the corresponding author)

1) I am aware of what I want for my future life

2) I’m spending a great deal of time on actions related to my future goals

3) I have clear goals for what I would like to achieve in life

4) I’m making efforts to achieve what I want for the future

5) I have a clear idea about the person I wish to be in the future

6) I have already begun to enact my plans for the future

7) I have already decided what to do with my life in the future

8) I’m engaged in activities to achieve my future goals

**Appendix 3**

Multivariate normality

|  | Unemployed individuals | | | | Workers | | | | Entire sample | | | |
| --- | --- | --- | --- | --- | --- | --- | --- | --- | --- | --- | --- | --- |
|  | Skewness | | Kurtosis | | Skewness | | Kurtosis | | Skewness | | Kurtosis | |
|  | M | p | M | p | M | p | M | p | M | p | M | p |
| FTOS | 442.9 | .000* | 12.2 | .000* | 625.1 | .000* | 19.2 | .000* | 903.8 | .000* | 25.2 | .000* |
| LPS | 550.1 | .000* | 21.5 | .000* | 532.4 | .000* | 19.1 | .000* | 837.2 | .000* | 32.6 | .000* |

*Notes. n* = 176, for unemployed individuals; and *n* = 176, for workers, *significant at *α* = .05, **Significant at *α* = .001, FTOS = Future Time Orientation Scale, LPS = Life Project Scale

**Appendix 4**

Statistical assumptions for MANOVA

|  | Multicollinearity | | | | | Shapiro-Wilk test | | Levene Test | | | | | | | | | | | | | |
| --- | --- | --- | --- | --- | --- | --- | --- | --- | --- | --- | --- | --- | --- | --- | --- | --- | --- | --- | --- | --- | --- |
|  |  |  |  |  |  |  |  | work. st. | | gender | | education | | int1 | | int2 | | int3 | | int4 | |
|  | Imp | Idn | Inv | Tol | VIF | W | p | Z | p | Z | p | Z | p | Z | p | Z | p | Z | p | Z | p |
| Dis | .27** | .11* | .14* | .92 | 1.08 | 0.94 | .00** | 0.05 | .83 | 1.17 | .28 | 0.18 | .67 | 0.44 | .73 | 0.25 | .86 | 0.58 | .63 | 0.45 | .87 |
| Imp |  | .24** | .33** | .84 | 1.19 | 0.96 | .00** | 0.03 | .86 | 1.46 | .23 | 0.07 | .79 | 1.68 | .17 | 0.05 | .99 | 0.69 | .56 | 1.19 | .31 |
| Idn |  |  | .76* | .42 | 2.41 | 0.93 | .00** | 4.10 | .04 | 6.33 | .01* | 0.00 | .98 | 3.66 | .01* | 3.69 | .01* | 2.32 | .08 | 2.72 | .01* |
| Inv |  |  |  | .39 | 2.54 | 0.95 | .00** | 0.03 | .83 | 1.46 | .23 | 0.07 | .79 | 1.68 | .17 | 0.05 | .99 | 0.69 | .56 | 1.19 | .31 |

*Notes.* *N* = 352, *p < .05, ** p < .001, work. st. = working status, int1 = interaction of working status and gender, int2 = interaction of working status and education, int3 = interaction of gender and education, int4 = interaction of working status, gender, and education, Dis = distance, Imp = impact, Idn = identification, Inv = involvement. Mardia’s test: M(skewness) = 237.9, p > .001, M(kurtosis) = 9.6, p < .001. Box’s M test: M = 129.0, p < .001.
